# Immunotherapy biomarkers 2016: overcoming the barriers

**DOI:** 10.1186/s40425-017-0225-6

**Published:** 2017-03-21

**Authors:** James L. Gulley, Jay A. Berzofsky, Marcus O. Butler, Alessandra Cesano, Bernard A. Fox, Sacha Gnjatic, Sylvia Janetzki, Shyam Kalavar, Vaios Karanikas, Samir N. Khleif, Ilan Kirsch, Peter P. Lee, Cristina Maccalli, Holden Maecker, Jeffrey Schlom, Barbara Seliger, Janet Siebert, David F. Stroncek, Magdalena Thurin, Jianda Yuan, Lisa H. Butterfield

**Affiliations:** 1 Genitourinary Malignancies Branch, Center for Cancer Research, NCI, 10 Center Dr., 13 N240, Bethesda, MD 20892 USA; 2 Vaccine Branch, Center for Cancer Research, 41 Medlars Dr, Bldg 41 Rm D702D, Bethesda, MD 20892 USA; 3 Princess Margaret Cancer Center/Ontario Cancer Institute, RM 9-622, 610 University Ave, Toronto, ON Canada; 4 NanoString, Inc., 500 Fairview Avenue North, Seattle, WA 98109 USA; 5 Earle A. Chiles Research Institute, Providence Cancer Center, 4805 NE Glisan Street, Portland, OR 97213 USA; 6 Department of Hematology/Oncology, Tisch Cancer Institute, Icahn School of Medicine at Mount Sinai, S5-105, 1470 Madison Avenue, Box 1128, New York, NY 10029 USA; 7 ZellNet Consulting, Inc., 555 North Avenue, Fort Lee, NJ 07024 USA; 8 Center for Devices and Radiological Health, U.S. Food and Drug Administration, 1401 Rockville Pike, Rockville, MD 20852 USA; 9 Roche Innovation Center Zurich, Wagistrasse 18, Schlieren, Switzerland; 10 Georgia Cancer Center, Augusta University, 1120 15th Street, CN-2101A, Augusta, GA 30912 USA; 11 Adaptive Biotechnologies, Inc., 1551 Eastlake Ave. E., Seattle, WA 98102 USA; 12 Department of Immuno-oncology, City of Hope, 1500 East Duarte Road, Duarte, CA 91010 USA; 13 Department of Translational Medicine, Sidra Medical and Research Center, Doha, Qatar; 14 Stanford University Medical Center, 299 Campus Drive, Stanford, CA 94303 USA; 15 National Cancer Institute, National Institutes of Health, 10 Center Drive, Bldg. 10, Room 8B09, Bethesda, MD 20892 USA; 16 Institute of Medical Immunology, Martin Luther University Halle-Wittenberg, Magdeburger Str. 2, Halle, Germany; 17 CytoAnalytics, 3500 South Albion Street, Cherry Hills Village, CO 80113 USA; 18 Department of Transfusion Medicine, National Institutes of Health, 10 Center Drive, Building 10, Room 3C720, Bethesda, MD 20892 USA; 19 National Cancer Institute, Cancer Diagnosis Program, DCTD, National Institutes of Health, 9609 Medical Center Drive, Bethesda, 20892 MD USA; 20 Early Clinical Oncology Development, Merck Research Laboratories, Rahway, NJ 07065 USA; 21 Department of Medicine, Surgery and Immunology, University of Pittsburgh Cancer Institute, 5117 Centre Avenue, Pittsburgh, PA 15213 USA

**Keywords:** Biomarkers, Immunotherapy, Immune monitoring, High throughput, Baseline measures, Tumor microenvironment

## Abstract

This report summarizes the symposium, ‘Immunotherapy Biomarkers 2016: Overcoming the Barriers’, which was held on April 1, 2016 at the National Institutes of Health in Bethesda, Maryland. The symposium, cosponsored by the Society for Immunotherapy of Cancer (SITC) and the National Cancer Institute (NCI), focused on emerging immunotherapy biomarkers, new technologies, current hurdles to further progress, and recommendations for advancing the field of biomarker development.

## Introduction

Dr. James L. Gulley and Dr. Lisa H. Butterfield opened the meeting with an introduction. The field of cancer immunotherapy has enjoyed unprecedented successes due to positive and durable clinical trial outcomes, the development of new drugs, biologics and combination therapies, and the approval of immunotherapeutics such as immune checkpoint inhibitors. Despite this progress, the current patient response rates and toxicities associated with immunotherapies have created a sense of urgency to determine which patients would most benefit from the treatment option. The identification of immune biomarkers will help to fill knowledge gaps by providing valuable predictive and prognostic information, and insights on the mechanisms that underlie patient responses or resistance to immunotherapy. This would allow development of treatment plans specific to each patient, which could help avoid selection of ineffective therapies, toxicities, and the resulting need to treat these toxicities. Furthermore, the rational design of combination therapies only becomes possible once mechanisms of action and resistance are fully elucidated.

Among current hurdles to the identification and development of clinically relevant biomarkers is the lack of standardized conditions for the collection and storage of specimens. Indeed, the data obtained from variably banked specimens are inconsistent, and at present, many trials bank only non-viable tumor and/or serum samples. The costs associated with immune assays can also be prohibitive, as robust and reproducible signals cannot be obtained by testing small numbers of samples, and attempts to keep costs low by focusing on one or two ‘best guess’ assays could lead investigators to miss the most accurate biomarker.

The availability of novel technologies and high throughput approaches (e.g. mass cytometry, whole exome sequencing, gene expression profiling, TCR diversity, epigenetics, etc.) offers unique opportunities and challenges for the field of biomarker development. Through the use of these powerful techniques, a multitude of questions can be addressed with a single sample, yet the resulting quantity and complexity of data require unique analytical considerations. The field of immune monitoring is also influenced by the discovery that metabolic considerations, the microbiome, and signaling pathway modulation all affect the immune system. Indeed, many standard of care therapies are now known to operate through immune mechanisms.

The SITC Biomarkers Task Force was convened to address the current state of the field, and recommend avenues for progress. The ‘Immunotherapy Biomarkers 2016: Overcoming the Barriers’ symposium focused on outcomes of topics addressed by four Task Force Working Groups. Divided between working groups (WGs), the following themes were discussed: Immune monitoring, assay standardization and validation (WG1); New developments in biomarker assays and technologies (WG2); Systematic assessment of immune regulation and modulation (high throughput approaches) (WG3); and Baseline immunity, tumor immune environment and outcome prediction (WG4). This report summarizes the outcomes from the meeting.

## Immunologic monitoring, assay standardization and validation (WG1)

WG1 was introduced by Dr. Magdalena Thurin. Putative biomarkers predictive of response to immunotherapy must undergo high quality and rigorous validation. As a result, very few biomarkers are approved as clinically useful. A major hurdle is that immune modulation affects many cell types and complex interactions while many drugs and small molecule inhibitors target single proteins or pathways. Greater understanding of the complexity of the validation process, especially for markers based on immune response, is needed in order to build up the resources available for immune monitoring. The two volume white papers from WG1, in which the analytical and clinical validation process, and regulatory considerations are discussed in depth, have been published [1, 2].

Dr. Alessandra Cesano presented an overview of the challenges faced in translating biological data into predictive biomarkers and described the example of Prosigna as a cleared diagnostic. A combination of factors from the host, cancer cells, and tumor microenvironment (TME), including the microbiome, shape interactions between the tumor and the immune system. This presents various hurdles, the first being the complexity of integrating these interactions with an informative analyte (DNA, RNA, and proteins) to yield a multivariate, multi-analyte assay. Although tens of thousands of markers in the GVK Biosciences (GVK Bio) Online Clinical Biomarker Database (GOBIOM) developed in collaboration with the U.S. Food and Drug Administration (FDA) have been identified, only a fraction of these have been developed into validated genomic biomarkers for FDA-approved drugs, and none have become multiplex, in vitro companion diagnostics. For a predictive biomarker to be applied in the clinic, it must have analytic (the ability to accurately and reliably measure analytes of interest in clinical specimens representative of the target population) and clinical validity, in addition to clinical utility. Multiple organizations have published guidelines for the validation of diagnostic tests, with recommendations regarding analytic sensitivity, specificity, reproducibility, and assay robustness [3, 4]. Dr. Cesano suggested that the co-development of drugs and companion diagnostics could address the biomarker deficiency. Although such an approach would increase the complexity of drug development, it may make the process more successful, and has great potential value for patients and health system resource utilization.

Dr. Ilan Kirsch detailed the capabilities of high-throughput PCR-based sequencing technology for T cell receptor (TCR) clonality assessment. The basis of this technology arose from the fact that the genes that encode functional immune receptors initially exist in germline DNA as a number of discrete, non-contiguous segments (variable, V, diversity, D, and joining, J). As a prerequisite to the formation of a functional immune receptor, a site-specific DNA breakage and rejoining event occurs that unifies any combination of these segments into a contiguous V(D)J region, which encodes the hypervariable part of the immune receptor. The assay uses forward primers from the V segments, and reverse primers from the J segments, to amplify and then sequence the area of this rearrangement event, thus creating a unique “barcode” of the lymphocyte and all clonal progeny of the rearranged cell. In this way, immunosequencing can quantify and specify every B and/or T cell in a sample of interest with a high degree of sensitivity and reproducibility. The superior accuracy of immunosequencing has been demonstrated in side-by-side comparisons with multi-parameter flow cytometry, in which immunosequencing successfully detected minimal residual disease in samples from patients with both T and B cell hematologic malignancies that were missed by flow cytometric techniques [5]. Additional applications of high-throughput clonality assessment include insights into drug mechanisms, measurements of immune system dynamics, and prognostic potential. In a study using immunosequencing to assess the number and clonality of tumor-infiltrating lymphocytes (TIL) from tissue samples of stage II DNA mismatch repair-proficient colon cancer patients, it was found that patients with below-median clonality and TIL number were at higher risk for disease recurrence [6]. This type of TIL assessment also may predict response to therapy. When applied to the setting of melanoma, patient samples with TIL beneath the median number and level of clonality were less likely to respond to anti-PD-1 therapy [7].

Dr. Sylvia Janetzki discussed challenges associated with single cell functional immune assay validation. The inherent variability associated with assays such as ELISPOT and intracellular cytokine staining [8], due to either the assay itself or due to the sample, needs to be considered. Sample quality must be addressed in pre-analytical validation and many guidelines already exist regarding peripheral blood mononuclear cell (PBMC) processing [9], avoidance of granulocyte contamination, apoptosis assessment, and overnight resting of samples. Assay variability arises from the lack of standardized protocols as well as the absence of a “gold standard”, which makes accuracy impossible to determine. To address the issue of assay variability, participation in proficiency panels [10] is recommended, as they provide an analysis of one’s own measurement in relation to measurements from many other groups in the field using many different protocols. Open proficiency panel programs already exist for ELISPOT and multimer panels and are available to all labs on a non-profit basis. In addition to evaluating the relative accuracy of data, proficiency panels can be used to identify critical protocol variables that are predictive of assay performance. These critical variables have been collected and summarized in published harmonization guidelines to be used by labs for optimization of assay outcomes [11], and are now available for ELISPOT assay and analysis, multimer staining, intracellular cytokine staining (ICS) assay, and gating. Finally, as precision is the most important parameter to ensure confidence in data analysis, intra-assay, inter-assay, and inter-operator precision need to be demonstrated in the form of repeatable results with a low false positive rate once assays have been harmonized.

Dr. Shyam Kalavar from the U.S. FDA presented on regulatory considerations for in vitro diagnostic devices in cancer immunotherapy. Companion in vitro diagnostic (IVD) devices provide information that is essential for the safe and effective use of a corresponding therapeutic product (e.g. PD-L1 IHC 22C3 pharmDx diagnostic test manufactured by Dako A/S (now Agilent Technologies)). In contrast, complementary diagnostics are tests that identify a biomarker-defined subset of patients that is expected to respond particularly well to a drug, and aid risk/benefit assessments for individual patients, but are not pre-requisites for receiving the drug (i.e., are not companion diagnostics). An example of a complementary IVD device is PD-L1 IHC 28–8, manufactured by Dako A/S (now Agilent Technologies) for non-squamous NSCLC and melanoma. As commercialization of IVD requires FDA clearance, a key regulatory question is whether adequate analytical and clinical performance data are available to be approved contemporaneously with the drug approval. In the case of immunohistochemistry-based assays such as PD-L1 IHC 22C3, analytical validation studies include biochemical characterization of the antibody, tests of assay sensitivity and specificity, precision, inter-laboratory reproducibility, assay robustness, the impact of pre-analytical variables, stability, and control cell line validation. Additional information regarding IVD regulation is available in guidance documents provided by the FDA to help direct validation of such devices.

Each section of the meeting had time for a panel discussion. The speakers addressed several questions, including those relating to publication of assay details for proper evaluation by the field.

## New developments in biomarker assays and new technologies (WG2)

Dr. Jianda Yuan introduced the WG2 areas of focus. Over the past year, the members of Biomarkers WG2 have collaborated to evaluate new technologies. These include quantitative real-time PCR-assisted cell counting (qPACC) [12], protein microarray (‘seromics’) [13], flow and mass cytometry [14], multiplexed tissue biomarker imaging [15], and whole exome sequencing for mutation load/neoantigen discovery [16]. All the resulting papers have been published in the Journal for ImmunoTherapy of Cancer (JITC) technology primer series [17–23], with still more on the horizon. Additionally, the WG2 white paper highlights novel technologies and emerging biomarkers relevant to individualized cancer immunotherapy, with recommendations for best practices unique to each [24].

As the prognostic power of TILs continues to gain widespread recognition [25, 26], Dr. Bernard A. Fox believes that multispectral imaging is critical to advancing our understanding of the complex interactions in the TME. Using markers of immune cell subsets, phenotype, and function [27], standardized multispectral immunohistochemistry is ideally suited to address interactions between multiple parameters, especially when patient sample availability is a consideration. However, difficulties associated with this technique include high background fluorescence, availability of antibodies, photobleaching, and image analysis [28]. The development of a tyramide signal amplification reagent was a breakthrough in multispectral technology that obviates such issues. This technique was used to interrogate tumors from a cohort of patients with melanoma to determine that the CD8:FoxP3 ratio predicts ability to generate TIL, the predictive power for which increases with the addition of PD-L1 [29]. The examination of multiple parameters thus offers value compared to single parameter analysis in a complex environment where ratios, relative positions, and functional analyses are mechanistically important. Dr. Fox suggested that advancements in multispectral imaging technology could provide support for stratification of patients for clinical trials in the future.

Dr. Jianda Yuan described how next-generation sequencing technologies such as whole exome sequencing are paving the way for precision oncology. Mutation-derived antigens (neoantigens) have long been an area of research interest, but the patient-specific nature of neoantigens represented a hurdle to the broader application of these data for a therapeutic purpose. The advent of whole exome sequencing has allowed for a comprehensive description of the mutation load, termed the “mutational landscape”, which has been a valuable resource to the field of immunotherapy, since neoantigen immunogenicity has been shown to be a correlate of response to treatment. For example, neoantigen-specific T cell reactivity was demonstrated via tumor exome sequencing in ipilimumab-responsive melanoma [30], and most patients who responded to ipilimumab treatment had a relatively high mutation load [31]. Similarly, the nonsynonymous mutation burden was associated with clinical benefit of anti-PD-1 therapy in patients with NSCLC [32], and MMR deficiency in colorectal cancer correlated with better clinical response to PD-1 blockade [33]. Potential uses of whole exome sequencing for neoantigen discovery and precision oncology include: prediction of patient response to vaccine and cancer immunotherapy, identification of new targets, therapeutic neoantigen vaccination, adaptive neoantigen T cell transfer therapy, and the design of effective combination immunotherapies. Strategies to move the field forward include combining approaches that integrate function (e.g. ELISPOT), phenotype (e.g. multicolor flow cytometry), and signature (e.g. whole exome sequencing, gene expression profile).

Dr. Holden Maecker spoke of the need to systematically measure immunocompetence in cancer patients, as traditional radiation and chemotherapy can be immunosuppressive, and immunotherapeutic agents interact directly with the immune system. Such an assessment of circulating immune subsets and functions could be prognostic of an overall response or a response to specific agents [34]. Mass cytometry (CyTOF) is a powerful alternative to fluorescence flow cytometry that can simultaneously assess multiple parameters with little to no spillover between channels. Dr. Maecker described two studies that employed the use of CyTOF technology to interrogate the immune systems of cancer patients at various time points, both of which suggested that specific immune profiles may be prognostic for response to immunotherapy. In particular, the presence of CD8+ central memory T cells and/or IL-2-producing T cells were associated with response to treatment. This immune profile may be a common predictor across multiple chronic conditions, as lack of CD8+ T cells that make IL-2 has been associated with disease progression in HIV [35]. The mTOR pathway might represent a means of boosting the central memory compartment in order to improve response rates for immunotherapies, as mTOR inhibition has been shown to reduce PD-1 expression and increase central memory T cells [36, 37].

Multifaceted immunomonitoring techniques to identify biomarkers predictive of clinical outcome were addressed by Dr. Cristina Maccalli. To capture the complexity of tumor-immune cell interactions, a multifaceted approach integrates different techniques for assessing immune cell phenotypes, functional capabilities, and negative immunoregulatory factors within the same patient sample. Such an approach was applied in a multicenter, controlled study of patients with melanoma being treated with an anti-CTLA-4 agent plus chemotherapy [38, 39]. Initially, standardized multiparameter flow cytometry was used for phenotype analysis of peripheral blood mononuclear cells (PBMC) to identify different subsets of T cells, NK cells, and B cells both at baseline and treatment. Soluble serum factors such as secreted natural killer group 2D (NKG2D) receptor ligands, which are upregulated in tumor cells and can impair T cell responses were also measured. These data were integrated with classification and regression trees (CART) and an interesting finding emerged, in which patients with longer overall survival were lacking the soluble ligand and had an increase in the frequency of CD4+ BTLA+ memory T cells and Th17-like cells at baseline. Further investigation with functional assays revealed a correlation between antigen-specific T cell responses and clinical outcomes [40]. Thus, multifaceted immunomonitoring platforms can help identify a functional immunological signature predictive of clinical outcome to immunotherapy, although this platform needs to be optimized and validated with a large cohort of patients undergoing immunotherapy treatments. Dr. Maccalli concluded by stating that the contemporaneous analysis of tumor and peripheral blood could define whether i) a specific tumor immunophenotype is characterized by a distinct peripheral blood gene signature and ii) those signatures correlate with clinical outcome. If it proves that gene signatures correlate with a peripheral blood cell subset phenotype, this would allow a detailed characterization of specific immunophenotype associated with patients’ clinical outcome.

The WG2 section had a panel discussion with the speakers. Topics discussed included the timing of sample testing, analysis of primary tumors and metastases, and the extent to which technologies and approaches can be prioritized based on existing data.

## Assessment of immune regulation and modulation systematically (high throughput approaches) (WG3)

Dr. David F. Stroncek presented the areas focused on by the third WG. WG3 sought to provide guidance on which tissue sources should be considered for analysis and monitoring during the process of clinical trial design. Sample materials discussed included serum/plasma, peripheral blood leukocytes, and the microbiome (a focus of the WG3 white paper). In addition, the means by which the tissue should be evaluated, such as proteomics, flow cytometry, gene expression, micro RNA expression, and mutation analysis approaches were considered, along with recommendations for data analysis. Adoptive cell therapies were also discussed. The rapid evolution of the science, and of analytical methods, as well as the very large quantities of data involved, present challenges to developing state-of-the-art guidance [41].

Dr. Peter P. Lee focused on three main aspects of the tumor microenvironment: 1) the importance of the tumor microenvironment to clinical outcomes, 2) the equal importance of the number of cells present and the spatial relationships between them, and 3) the tumor-draining lymph nodes (TDLN), which are the major site of cancer cell-immune cell interaction. Tumors comprise more than cancer cells alone, since roughly 50% of human tumors are >50% stroma. Patients with ‘stroma-rich’ tumors have poorer clinical outcomes because stromal cells provide growth and metabolic factors that protect cancer cells from a variety of anti-cancer drugs, including chemotherapeutic agents and targeted drugs such as BRAF inhibitors. Indeed, stromal cell density appears to be an independent prognostic marker in breast, colorectal, pancreatic, and other cancers. As mentioned by other presenters, intratumoral immune cells are also predictive of clinical outcome, which led to the Immunoscore effort [42] to aid in clinical decision-making. The interplay between cancer cells, immune cells, and non-immune stromal cells within the TME can be studied using an automated quantitative pathology imaging system. This system allows for the identification of spatial immune cell patterns within the tumor, which may have implications for clinical outcomes. For example, to address whether TDLN functionality is altered in cancer, immune cell populations were evaluated in disease-free versus relapsed breast cancer patients. A significantly higher percentage of CD4+ T cells and CD1a + dendritic cells (DC) was found in the draining lymph nodes of patients who remained disease-free versus those who had relapsed, and this immune profile alone correlated with disease-free survival [43]. Spatially, DC within lymph nodes of disease-free patients were found in clusters, whereas the architecture of DC clusters from the nodes of patients whose disease had relapsed were disrupted —a pattern that also correlated with clinical outcome [44]. Quantitative, spatial image analysis of tumors and TDLN will be highly informative in understanding how therapies modulate the balance between cancer and host immune responses.

The importance of monitoring adoptive cellular therapies as a means of improving clinical outcomes for patients was presented by Dr. David F. Stroncek. Multiple studies of tumor-infiltrating lymphocytes (TIL) as sources of autologous cells for transfer in patients with melanoma have yielded markers indicative of clinical outcome. Specifically, higher total numbers and percent of CD8+ T cells infused are associated with improved outcomes, as is persistence of T cell clones post-infusion. Other markers have been correlated with outcomes in individual studies and may merit additional investigation, such as telomere length and expression of CD27 [45]. Studies of CD19 CAR T cells in patients with lymphoma or acute lymphocytic leukemia (ALL) have demonstrated that peak levels of CAR T cells with a high CD8:CD4 T cell ratio, and higher numbers of CD8+ effector T cells, are associated with a better response [46–48]. Factors that influence high peak blood levels of CD19 CAR T cells include the presence of CD19+ cells at the time of infusion (lymphoma) [47] and quantities of blasts in bone marrow (ALL). Dr. Stroncek also commented on three complications associated with adoptive cellular therapies: tumor lysis syndrome, cytokine release syndrome (CRS), and neurotoxicity, and the use of biomarkers to assess them. Patients with a higher tumor burden and greater numbers of circulating CAR T cells are more likely to experience CRS [46]. In the clinic, elevated levels of IFNγ and IL-6, or C-reactive protein (CRP) as a surrogate for IL-6, can be tested for, as markers of toxicity [46]. Closing recommendations focused on monitoring levels of adoptively transferred cells; analyzing infused cells for phenotype, gene expression, and polymorphisms; and tracking CRP levels post-treatment.

Dr. Barbara Seliger opened the discussion about immune monitoring of clinical trials by noting that the primary goal of cancer immunotherapy is to increase anti-tumor activity by activating the immune response, altering the tumor microenvironment, and overcoming tumor-associated barriers. In order to analyze the efficacy of these strategies, it is necessary to integrate multiple high-throughput technologies, which requires investigators to obtain patient samples of peripheral blood, tumor cells, and tumor-infiltrating cells. Ideally, samples should be collected prior to, during, and after treatment, depending on the type of immunotherapy used. Although such an approach is best suited for peripheral blood and in the setting of melanoma, the discovery of links between the immune response and the expression of immune modulatory molecules on tumors may present alternative approaches to monitoring. For example, a decrease in tumor antigen expression is associated with a lack of tumor-specific immune response, and alterations in class I antigen presentation can lead to reduced effector cell cytotoxicity. [49]. It is also important to note that certain types of immunotherapy, including peptide vaccination, checkpoint inhibitors, and adoptive T cell transfer, have been shown to allow for the evolution of acquired resistance to treatment by selecting for HLA class I loss variants. HLA-G is a non-classical HLA class I molecule that is a putative novel inhibitory checkpoint, as it is often over-expressed in tumors, can inhibit NK and T cell mediated cytotoxicity, and is associated with poorer prognoses in a variety of solid tumors [50]. Dr. Seliger closed by saying that it is important to understand the mechanisms of immune escape in order to appropriately apply immunomodulatory agents, but immune monitoring should not cease once a therapy has been selected. Rather, continued monitoring is an important means of addressing resistance to treatment that may emerge over time.

Ms. Janet Siebert described the current state of immune monitoring as a compilation of “humpty dumpty data sets” in her presentation on Analysis of the Systemic Host Response. Her statement emphasized that contemporary data sets are generated through interrogation of multiple tissues, using multiple assays, from multiple organizations (from multiple intra- or inter-institutional cores), across academia, industry, and government. Such fragmentation could reduce the feasibility of measuring of the systemic response. Instead, the field needs to move toward the compilation of integrated heterogeneous data sets or “het sets”, which use a consistent assay-agnostic format that spans assays, tissues, and organizations, and supports analysis of the systemic cross-compartment response. Advantages of het sets include a common technical and conceptual representation of an otherwise unwieldy data set, the application of the same analytical tools to a large number of analytes from different assays, and the ability to apply established multivariable analytical approaches to the integrated whole. Ideally, these analytical approaches would be well-established and magnitude-insensitive with both visual and quantitative results that can be easily validated in follow-up studies. Decision tree classification is an example of a suitable analytical approach that seeks to determine which analytes most cleanly separate patients into responder and non-responder groups. These can be supplemented with reference data sets to ensure that thresholds are not arbitrary or over-fitted. To address systemic responses that span assays or tissues, linear regression modeling can be used to capture the relationship between analyte pairs [51]. The resulting complex immunological data can then be synthesized with a network arc diagram to provide succinct visualization, and insights into underlying the biological mechanisms of interest, such as responsiveness to therapy [52].

During the WG3 panel discussion, the speakers commented on the use of high throughput technologies to test adoptive cellular therapies, and detection of tertiary lymphoid structures.

## Prediction of clinical outcome based on baseline measures (WG4)

Dr. Sacha Gnjatic introduced the fourth WG and its activities. Aiming to provide recommendations on how to predict a patient’s response to treatment through analysis of baseline biomarkers in blood and tumor, including MDSC and other immature myeloid cells, and checkpoint molecule expression. WG4 also focused on the diversity of immune cells present in the TME (including tertiary lymphoid structures), effectiveness of peripheral surrogates of the TME, and new technologies to aid in the comprehensive analysis of baseline immunity (*manuscript in revision for the Journal for ImmunoTherapy of Cancer*).

Investigations into the tumor microenvironment at a genetic level, as presented by Dr. Samir N. Khleif, seek to address whether genetic changes within the tumor microenvironment can guide the design of cancer immunotherapeutics. The power of this approach was illustrated by investigations into Ras as a mutated antigen immune target. In contrast to prognostic or predictive biomarkers, immune targets are biomarkers that might not correlate strongly with response to treatment, such as PD-L1, but can help direct the development of therapies. Dr. Khleif described a study in which Ras mutations were used as immune target biomarkers. Patients with advanced solid tumors bearing Ras mutations were given a cancer vaccine comprised of autologous peptides along with IL-2, GM-CSF, or both. Although most patients developed antigen-specific immune responses, only one patient out of 57 generated productive immunity that went on to eliminate the tumor cells [53]. This disparity led to the discovery that regulatory T cell (Tregs) are significantly expanded in colon cancer patients with mutated Ras compared to both healthy individuals and colon cancer patients with wild-type Ras. It was found that mutant Ras activates the MEK-ERK-AP1 pathway to induce secretion of high levels of IL-10 and TGF-β1, which generate local induction of regulatory T cells (Treg) in the tumor microenvironment [54]. Ras mutation was noted to be an early event in the development of tumors leading to the proliferation of affected cells, and the resulting induction of Treg serves to support tumor immune escape by creating a suppressive microenvironment that inhibits the anti-tumor immune response. Dr. Khleif suggested that an additional agent targeting Treg could boost the efficacy of a cancer vaccine in patients with Ras mutations. Thus mutated antigens used as immune target biomarkers can also guide investigations into immune bystander effects on the microenvironment, with the potential to inform the development of rational combination treatments.

In a forward-looking discussion, Dr. Vaios Karanikas presented on multiplex immunohistochemistry (IHC) in clinically annotated material: where are we and where are we going? As more clinical data become available from patients treated with cancer immunotherapies, it has become clear that patients with inflamed tumors are more likely to respond to treatment. A deeper understanding of the interactions between the immune system and tumor cells within the tumor microenvironment is needed to achieve the ultimate goal of designing therapeutic strategies capable of inflaming “cold” tumors and addressing immune escape mechanisms. The identification of predictive and prognostic biomarkers using high throughput approaches like multiplex IHC would aid this endeavor and Dr. Karanikas offered a number of best practices for consideration. As tumor cells do not develop in isolation, it is imperative to assess the TME comprehensively (immune cells, stroma, and tumor cells), and compare paired samples from the same patient before, during, and after treatment. Choice of technology, e.g., chromogenic vs immunofluorescence, and antibody selection, can be guided by pragmatism. A relevant example of the complex questions that can be addressed by multiplex IHC includes the efforts to define the tumor-infiltrating cell types. This approach demonstrated the prognostic value of specific subsets of immune cell infiltrates [55, 56], their spatial distribution within the TME [57, 58], and qualitative characteristics such as an exhausted phenotype [7, 59]. Importantly, the integration of complementary technologies such as multiplex IHC, mutational profiling, and gene expression patterns can overcome the limitations of each method, as well as offer an improved understanding of the TME toward the identification of predictive and prognostic immune biomarkers.

The identification of biomarkers in the peripheral blood – the most readily available tissue for sampling – to assess a patient’s response to treatment was the focus of Dr. Marcus O. Butler’s Multiplex/Blood Profiles presentation. Central to this approach is a need to identify whether biomarkers in the TME are also present in the peripheral blood. Investigations into circulating PD-1+ lymphocytes, which have been shown to contain T cells specific for tumor-associated antigens and neoantigens, have provided evidence to suggest that this is possible, although such populations of interest are less frequent in blood [60]. The main tools available to interrogate rare cell populations include flow and mass cytometry and a number of different computational methodologies have been published to guide the analysis of these complex data [61]. In addition to its utility as a window into the TME, the peripheral compartment can be used to try to influence baseline immunity. One such study involved the adoptive transfer of in vitro-activated tumor antigen-specific T cells into patients with metastatic melanoma, the result of which was a stable increase in anti-tumor central memory cells detectable in the peripheral blood [62]. Upon subsequent treatment with anti-CTLA-4, the transferred cells expanded and generated partial responses [62] that laid the foundation for follow up studies including Adoptive Cell Therapy InVigorated to Augment Tumor Eradication (ACTIVATE): Cohorts 1–3.

In a discussion about B cells at the tumor site, and systemic humoral responses, as predictive biomarkers, Dr. Sacha Gnjatic noted that B cells organized in tertiary lymphoid structures (TLS) can be identified near tumors and the presence of dense follicular B cells along with mature DC is associated with a good prognosis in non-small cell lung cancer (NSCLC) [63]. Taking these observations a step further, investigators sought to determine whether the specificity of local and circulating antibodies may reflect the immunogenicity of the tumor. Tumor antigen-specific humoral immunity has been previously shown to correlate with CD4+ and CD8+ T cell responses to the cancer-testis antigen NY-ESO-1 [64, 65], and more recent studies in patients with NSCLC have gone on to demonstrate correlations between anti-NY-ESO-1 antibody titers and tumor stage, overall survival, and clinical course following anti-CTLA-4 immunotherapy [66, 67]. To translate humoral immune monitoring to the clinic, a seromics approach would allow for a sensitive and high-throughput means of detecting alterations in the antibody repertoire as a surrogate for the tumor immune response. Additional work in the area of comprehensive immune monitoring will need to establish appropriate targets that assign functionality to antigen specificity and relate to clinical outcomes.

The WG4 panel discussion addressed issues of tumor acquisition, including cost, logistics, and enabling a culture of routine tissue banking at different institutions and clinical settings.

## National cancer institute perspectives on biomarkers

In a presentation focused on lessons learned from the peripheral immunoscore, Dr. Jeffrey Schlom, discussed applications for the current state of flow-based technology that now allows for identification of 123 immune cell subsets from one tube of blood, using 30 markers. The subsets analyzed include nine standard immune cell subsets and 118 additional subsets relating to maturation and function [68]. In particular, monitoring of multiple peripheral immune subsets can aid in the identification of patients most likely to benefit from immunotherapy. For example, one study of patients with metastatic breast cancer treated with chemotherapy alone or in combination with cancer vaccine sought to use baseline measures of peripheral immunity to discriminate between patients with longer or shorter PFS. Indeed, a correlation between the presence of particular immune cells at baseline and disease progression was noted, although only in the combination therapy arm [69]. Such peripheral immunoscore analyses are intended to complement biopsy analyses, and can be used in many cancer types and stages for which biopsies are not easily obtained. Peripheral immune monitoring can extend beyond cells to the level of antigens. Several studies have demonstrated improved clinical outcomes due to the presence of cascade antigens (epitope spreading) following treatment with cancer vaccines as a result of tumor cell lysis and subsequent cross-priming [70–74]. For example, a trial using the cancer vaccine PROSTVAC in patients with prostate cancer revealed that a majority of patients generated T cells that mounted an immune response to antigens not present in the vaccine [75]. These data indicate a means by which heterogeneity among tumor cells can be minimized therapeutically, and Dr. Schlom suggested that all cancer vaccine clinical trials should include cascade antigen analyses.

Dr. Jay A. Berzofsky discussed the utility of extracellular vesicles (EV) as biomarkers of immune responses and tumor responses in cancer immunotherapy. Although previously underestimated as a homogeneous population, EV are gaining interest as diverse subsets that play many immunoregulatory roles in cancer. Released by both immune cells and tumor cells, EV are 100 nm particles capable of shuttling “packets” of information in the form of mRNA, miRNA, and protein between cells, the effects of which can be immunostimulatory or immunosuppressive. Similar to standard FACS technology, nanoFACS was developed to analyze, sort, and decode EV subsets based on light scatter and fluorescence. Once sorted, the EV could be further analyzed by molecular profiling via protein expression and RNA/DNA typing. The analysis of EV from patient plasma samples could provide a means of monitoring the immune response and directing treatment decisions, and it has the potential to make both membrane-bound and soluble (exosome-bound) TME-specific biomarkers accessible via “liquid biopsy”.

## Conclusions

In his closing remarks, Dr. James L. Gulley was optimistic that the opportunities presented by the use of biomarkers for immune monitoring outweighed current challenges. Having raised awareness of potential difficulties in immune biomarker research, the SITC Biomarkers Task Force WGs have also made recommendations for how to approach these challenges, and will continue to provide guidance as the field develops.

## Abbreviations

ALL: Acute lymphoblastic leukemia
CRS: Cytokine release syndrome
CyTOF: Cytometry by time-of-flight, mass cytometry
DC: Dendritic cell(s)
EV: Extracellular vesicle(s)
FDA: U.S. Food and Drug Administration
ICS: Intracellular cytokine staining
IHC: Immunohistochemistry
IVD: In vitro diagnostic
JITC: Journal for immunotherapy of cancer
NCI: National cancer institute
NKG2D: Natural killer group 2D
NSCLC: Non-small cell lung cancer
PBMC: Peripheral blood mononuclear cells
PFS: Progression-free survival
qPACC: Quantitative real-time PCR assisted cell counting
SITC: Society for immunotherapy of cancer
TCR: T cell receptor
TDLN: Tumor-draining lymph nodes
TIL: Tumor-infiltrating lymphocyte(s)
TLS: Tertiary lymphoid structures
TME: Tumor microenvironment
Treg: Regulatory T cell(s)
WG: Working group.
